# Multi-omics analysis reveals the role of ribosome biogenesis in malignant clear cell renal cell carcinoma and the development of a machine learning-based prognostic model

**DOI:** 10.3389/fimmu.2025.1602898

**Published:** 2025-06-26

**Authors:** Zhouzhou Xie, Shansen Peng, Jiongming Wang, Yueting Huang, Xiaoqi Zhou, Guihao Zhang, Huiming Jiang, Kaihua Zhong, Lingsong Feng, Nanhui Chen

**Affiliations:** ^1^ Affiliated Meizhou Hospital of Shantou University Medical College, Meizhou, China; ^2^ Department of Urology, Meizhou People’s Hospital, Meizhou Academy of Medical Sciences, Meizhou, China

**Keywords:** ribosome biogenesis, clear cell renal cell carcinoma, multi-omics analysis, malignant cells, machine learning, prognostic model

## Abstract

**Background:**

Clear cell renal cell carcinoma (ccRCC) is the most common subtype of renal cancer, marked by high molecular heterogeneity and limited responsiveness to targeted or immune therapies. Ribosome biogenesis (Ribosis), a central regulator of cell growth and metabolism, has emerged as a driver of tumor aggressiveness. However, its role in ccRCC pathogenesis and prognosis remains poorly defined.

**Methods:**

We integrated bulk RNA sequencing, single-cell RNA sequencing, and spatial transcriptomics sequencing data to dissect the biological functions and clinical relevance of Ribosis-related genes in ccRCC. Through pseudotime trajectory analysis and metabolic flux inference, we examined malignant progression and metabolic reprogramming. A prognostic model based on a Ribosis-related signature (RBRS) was built using 118 machine learning algorithm combinations and validated in internal and external cohorts. A web-based calculator was also developed. We further analyzed immune infiltration, genomic alterations, tumor microenvironment features, and drug sensitivity. Expression of five core Ribosis-related genes (RPL38, RPS2, RPS14, RPS19, RPS28) was validated by qRT-PCR.

**Results:**

We identified a Ribosis-high malignant subpopulation with enhanced stemness, poor prognosis, and elevated oxidative phosphorylation. These cells showed increased metabolic activity, especially in the pyruvate–lactate axis, potentially facilitating immune evasion. The RBRS model outperformed 32 published signatures (C-index = 0.68). High-risk patients exhibited an “immune-activated yet immunosuppressed” microenvironment, with increased CD8^+^ T-cell infiltration and elevated regulatory T cells, myeloid-derived suppressor cells, and immune checkpoint expression (e.g., PDCD1, CTLA-4). Despite active antigen presentation and immune cell recruitment, terminal tumor-killing capacity was impaired. High-risk tumors also showed higher mutation burden, frequent copy number loss of tumor suppressor genes, and resistance to common targeted therapies. The five RBRS genes were significantly upregulated in tumor tissues, consistent with bulk RNA-seq data.

**Conclusion:**

We reveal Ribosis as a key driver of ccRCC progression. The RBRS model demonstrates robust prognostic value and translational utility, linking Ribosis to metabolism, immune dysfunction, and therapy resistance, offering new insights for risk stratification and precision treatment in ccRCC.

## 1 Introduction

Renal cell carcinoma (RCC) is a globally prevalent malignancy, with an estimated 430,000 new cases diagnosed annually (1). Among its histological subtypes, clear cell renal cell carcinoma (ccRCC) is the most common, accounting for approximately 70–80% of all RCC cases (2). Due to its insidious onset and lack of early clinical symptoms, a substantial proportion of ccRCC patients are diagnosed at advanced stages, limiting the efficacy of conventional therapies. Nearly 50% of ccRCC cases develop distant metastases, and the 5-year overall survival (OS) rate for these patients remains as low as 14%, reflecting a poor prognosis (3). In recent years, significant advances have been made in targeted therapies and immunotherapy, particularly in the emergence of combination regimens such as pembrolizumab plus axitinib, which has become a first-line standard treatment for advanced ccRCC (4). Nevertheless, despite recent therapeutic advancements, the complex pathogenesis of ccRCC—marked by high intertumoral heterogeneity, the emergence of drug-resistant subclones, and multifaceted tumor–microenvironment interactions—continues to limit the clinical efficacy of current treatment strategies (5). Thus, the identification of robust biomarkers and molecular signatures is urgently needed to predict patient outcomes and guide treatment selection.

Ribosome biogenesis (Ribosis) is a fundamental cellular process essential for protein synthesis, involving the transcription and processing of ribosomal RNA (rRNA), assembly of ribosomal proteins, maturation of subunits, and nucleocytoplasmic transport (6). In cancer cells, this process is often dysregulated, characterized by increased pre-rRNA synthesis, aberrant rRNA modifications, and alterations in ribosomal proteins that give rise to so-called “onco-ribosomes.” These specialized ribosomes preferentially translate oncogenic mRNAs, contributing to functional reprogramming and metabolic adaptation that drive tumor progression (7). Aberrantly elevated Ribosis supports unrestricted cell proliferation and has been recognized as both a hallmark and a therapeutic vulnerability in multiple cancers (8, 9). For instance, the ribosomal methyltransferase SMYD5 promotes hepatocellular carcinoma by methylating lysine residues on RPL40, thereby enhancing translational activity (10). Similarly, suppression of SMYD5-mediated methylation of RPL40 was shown to inhibit growth in patient-derived xenograft models of gastric adenocarcinoma (11). Myelocytomatosis (MYC) oncogene is a key driver of ribosomal biogenesis, directly enhancing ribosomal DNA transcription and upregulating ribosomal proteins such as RPL14 and RPL28 to promote lymphomagenesis (12). Moreover, RAS signaling facilitates ribosome synthesis by phosphorylating nucleolin, enhancing its affinity for rRNA and accelerating the proliferation of pancreatic cancer cells (13).

**Figure 1:**
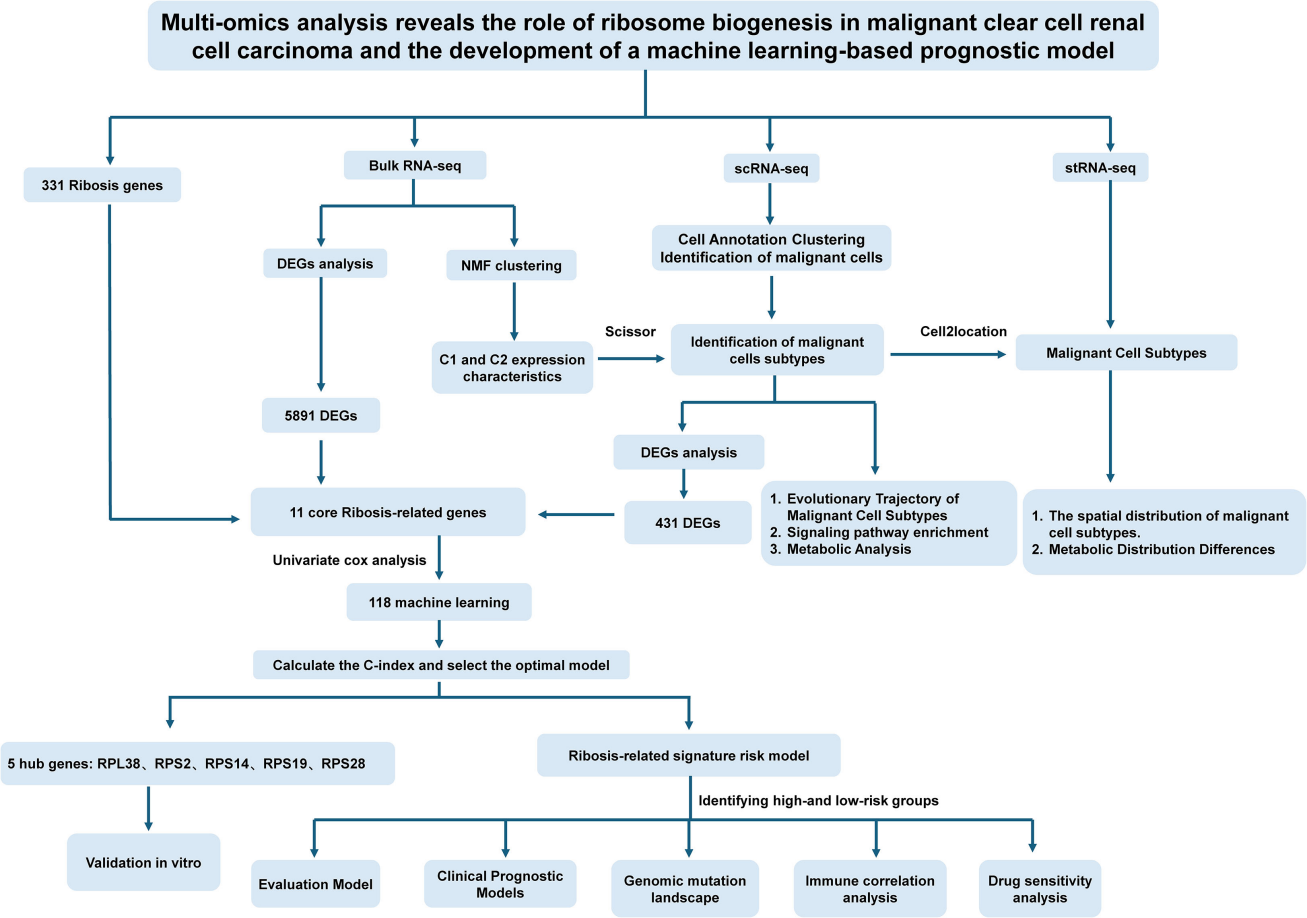
Schematic overview of the study design. Ribosis, ribosome biogenesis; Bulk RNA-seq, bulk RNA sequencing; scRNA-seq, single-cell RNA sequencing; stRNA-seq, spatial transcriptomics RNA sequencing; DEGs, differentially expressed genes; NMF, non-negative matrix factorization; C-index, concordance index; C1, cluster 1; C2, cluster 2.

Recent advances in understanding ribosome specialization and the complexity of Ribosis have unveiled previously unrecognized opportunities for the development of ribosome-targeted therapies in cancer (14). Despite growing evidence linking aberrant Ribosis to tumorigenesis, the molecular characteristics and clinical significance of Ribosis-related genes in ccRCC remain largely unexplored. To address this gap, we conducted an integrative multi-omics analysis to delineate the expression dynamics and metabolic dependencies of Ribosis-related genes in malignant ccRCC cells. Furthermore, we constructed a prognostic model—ribosome biogenesis-related signature (RBRS)—using machine learning algorithms, and comprehensively investigated its association with genomic alterations, tumor immune microenvironment, and therapeutic response. We also developed a web-based RBRS calculator (https://drxie2018510136.shinyapps.io/shinyapp/) to facilitate clinical translation. A schematic overview of our study design is presented in **Figure 1**.

## 2 Materials and methods

### 2.1 Data acquisition

Bulk RNA sequencing (bulk RNA-seq) and corresponding clinical data of ccRCC patients were obtained from The Cancer Genome Atlas (TCGA, https://portal.gdc.cancer.gov/), including 522 tumor samples and 72 adjacent normal samples, after excluding those with incomplete survival information. An independent validation cohort (E-MTAB-1980), comprising 101 ccRCC samples, was retrieved from the ArrayExpress database (https://www.ebi.ac.uk/arrayexpress/). Single-cell RNA sequencing (scRNA-seq) data were derived from the GSE210038 dataset (n=7 tumor samples) via the Gene Expression Omnibus (GEO) database. Spatial transcriptomics RNA sequencing (stRNA-seq) was acquired from the “TJ-RCC” cohort (15) (samples “R114T” and “Z43T”) available through ZENODO (https://zenodo.org/records/8063124). Ribosis-related genes were curated from a recent comprehensive study (8), yielding a total of 331 genes (**Supplementary Table S1**).

### 2.2 Preprocessing of RNA-seq data

Raw count matrices from TCGA were used for differential gene expression analysis using “DESeq2” R package (16). Transcripts per million (TPM) values were extracted from all cohorts for downstream analyses, and genes with mean TPM < 0.1 were excluded. scRNA-seq data were processed using the Seurat R package (17), employing standard quality control filters: genes expressed in 3+ cells and within 250–4000 gene count range, total unique molecular identifiers > 500 per cell, mitochondrial content < 10%, hemoglobin genes < 0.1%, and ribosomal gene quality > 3%. After principal component analysis (PCA) using the top 2,000 variable genes, batch effects were corrected using Harmony (18), and clustering was performed using FindNeighbors and FindClusters functions. Cells were visualized via Uniform Manifold Approximation and Projection (UMAP) and annotated using canonical marker genes.

### 2.3 Integration of spatial and single-cell transcriptomes

We employed “cell2location” Python package (19) to map scRNA-seq-identified cell types onto the spatial transcriptomic data. A negative binomial regression model was used to estimate cell-type-specific gene expression at each spatial location, with N_cells_per_location = 30 and detection_alpha = 20.

### 2.4 Identification of malignant cells and Ribosis phenotypes

Malignant epithelial cells were distinguished from non-malignant counterparts using the “CopyKAT” R package (20), which infers chromosomal copy number variation (CNV) from single-cell transcriptomes. Ribosis transcriptional phenotypes in TCGA-ccRCC were determined using non-negative matrix factorization (NMF) (21). The “Scissor” R package (22) was applied to integrate bulk RNA-seq phenotypes with single-cell data, enabling identification of phenotype-specific malignant cell subpopulations.

### 2.5 Pseudotime trajectory and stemness analysis

“Monocle” R package (23) was used to reconstruct the developmental trajectories of epithelial and malignant cells. The “CytoTRACE” R package (24) assessed stemness scores to inform lineage hierarchies. “ClusterGVis” R package (https://github.com/junjunlab/ClusterGVis) enabled unsupervised clustering and visualization of temporally regulated genes along the pseudotime axis.

### 2.6 Single-cell metabolic pathway analysis

To investigate the metabolic heterogeneity across different cell subpopulations in the scRNA-seq data, we employed the “SCPA” and “scMetabolism” R packages for evaluation (25, 26). The “SCPA” algorithm is suitable for comparing metabolic differences between cell clusters, but it does not provide metabolic activity scores at the single-cell level. Therefore, we used the “scMetabolism” algorithm in parallel to quantify enrichment scores of target metabolic pathways, enabling cross-validation of results. A curated high-confidence metabolic gene set compiled by Wu et al. (26) was used as the reference for all analyses (**Supplementary Table S2** for details).

### 2.7 Metabolic flux and metabolite abundance analysis

We applied the “scFEA” R package, which leverages graph neural networks to infer intracellular metabolic fluxes and metabolite abundances from scRNA-seq data (27). A reference set consisting of 168 human metabolic modules and 70 metabolites was used to construct a directed factor graph, where each metabolic module is represented as a factor node and each intermediate metabolite as a variable node, with flux balance modeled via a likelihood function. After computing fluxes and metabolite levels, we visualized metabolic differences across cell types using heatmaps based on scRNA-seq data and mapped the spatial distribution of metabolic activity onto histological slices from the stRNA-seq data.

### 2.8 Differential Ribosis-related gene expression

Differentially expressed genes (DEGs) between tumor and normal samples in TCGA were identified using “DESeq2” with |log2FC| > 1 and adjusted P < 0.05 (16). Within scRNA-seq data, DEGs were identified between Scissor (+) and Scissor (–) malignant cells using “FindMarkers” function (|log2FC| > 0.5, adjusted P < 0.05). The intersection of DEGs with Ribosis-related genes was taken as candidate genes for model construction.

### 2.9 Machine learning–based prognostic signature development

Univariate Cox regression was performed on intersected Ribosis-related genes to identify survival-associated candidates. The TCGA-ccRCC cohort was randomly split (1:1) into training and test sets using the “caret” R package, and the E-MTAB-1980 cohort was used for external validation. Based on the study by Liu et al. (28), we constructed a prognostic signature for Ribosis-related genes by systematically testing 118 algorithmic combinations derived from 10 machine learning approaches. These included “random survival forest”, “elastic net”, “Lasso”, “Ridge”, “Stepwise Cox regression”, “CoxBoost”, “partial least squares regression for Cox”, “supervised principal components”, “generalized boosted regression modeling”, and “survival support vector machine”. The best model was selected based on the highest average concordance index (C-index) in the test and validation sets. The final RBRS model formula is:

Risk score = Σ (coef_i × expression_i)

where coef_i is the gene’s Cox coefficient and expression_i its expression level.

### 2.10 Clinical survival analysis and nomogram construction

Patients were stratified into high- and low-risk groups based on the median RBRS score. Kaplan–Meier survival and receiver operating characteristic (ROC) analyses were performed to evaluate the model’s discriminatory ability across datasets. The independent prognostic value of RBRS was assessed using Cox proportional hazards regression. A nomogram was constructed by integrating RBRS with clinical features, and its performance was evaluated using calibration plots, C-index, ROC curves, and decision curve analysis (DCA). An interactive web-based tool was developed using the “shiny” website (https://www.shinyapps.io/) to facilitate clinical implementation of the prognostic model.

### 2.11 Genomic mutation and copy number analysis

Somatic mutation and CNV data were retrieved from “UCSC Xena” website (https://xena.ucsc.edu/). Tumor mutation burden (TMB) and intratumoral heterogeneity were assessed using “maftools” R package (29). TMB scores were calculated as the number of somatic nonsynonymous or total mutations per megabase within the whole exome or targeted sequencing regions of tumor samples, which is of critical importance for evaluating tumor heterogeneity (30). Stratified survival analysis was performed based on TMB and RBRS. Mutation landscapes and CNV profiles were compared across RBRS groups.

### 2.12 Functional and enrichment analysis

ClusterProfiler (31) was used for GO (Gene Ontology), KEGG (Kyoto Encyclopedia of Genes and Genomes), and GSEA (Gene set enrichment analysis) enrichment of DEGs. “AUCell” (24) and “GSVA” (32) R packages quantified pathway activity in scRNA-seq and bulk RNA-seq data, respectively. Gene sets were obtained from the Molecular Signatures Database (33).

### 2.13 Immune microenvironment and checkpoint analysis

Immune cell infiltration was quantified using “ssGSEA” (34) and “CIBERSORT” R packages (35). “ESTIMATE” (36) scores were used to assess immune/stromal content and tumor purity. Tumor Immuno Phenotype (TIP) analysis (37) was performed to assess anti-tumor immunity across seven immunological steps. Expression of immune checkpoints was compared across RBRS groups.

### 2.14 Drug sensitivity and in silico drug prediction

Drug sensitivity prediction was performed using “oncoPredict” R package (38). ConnectivityMap (cMap) analysis (https://clue.io/) was used to identify small molecules capable of reversing RBRS-associated gene expression signatures.

### 2.15 Quantitative real-time polymerase chain reaction validation

Expression levels of the five RBRS genes were validated via qRT-PCR in 12 paired tumor and adjacent normal tissues collected from Meizhou People’s Hospital (October 2024–January 2025). RNA was extracted using TRIzol™ (HaiGene, Harbin, China), and cDNA synthesis was performed using PrimeScript RT (RR047A, Takara, Dalian, China). qPCR was conducted using TB Green Premix Ex Taq II (RR802A, Takara, Dalian, China) on a 7500 ABI platform (Thermo Fisher Scientific Inc.) under standard cycling conditions. Relative expression levels were quantified using the 2^–ΔΔCt method (39) (primer sequences in **Supplementary Table S4**).

### 2.16 Statistical analysis

All analyses were performed using R (v4.4.1) and Python (v3.10). Two-group comparisons were assessed using Student’s t-test or Wilcoxon test. ANOVA was used for multi-group comparisons. Categorical variables were analyzed via Chi-square or Fisher’s exact test. Survival was analyzed using Kaplan–Meier and log-rank tests. Statistical significance was set at P < 0.05.

## 3 Results

### 3.1 Identification of malignant cells from scRNA-seq data

**Figure 2:**
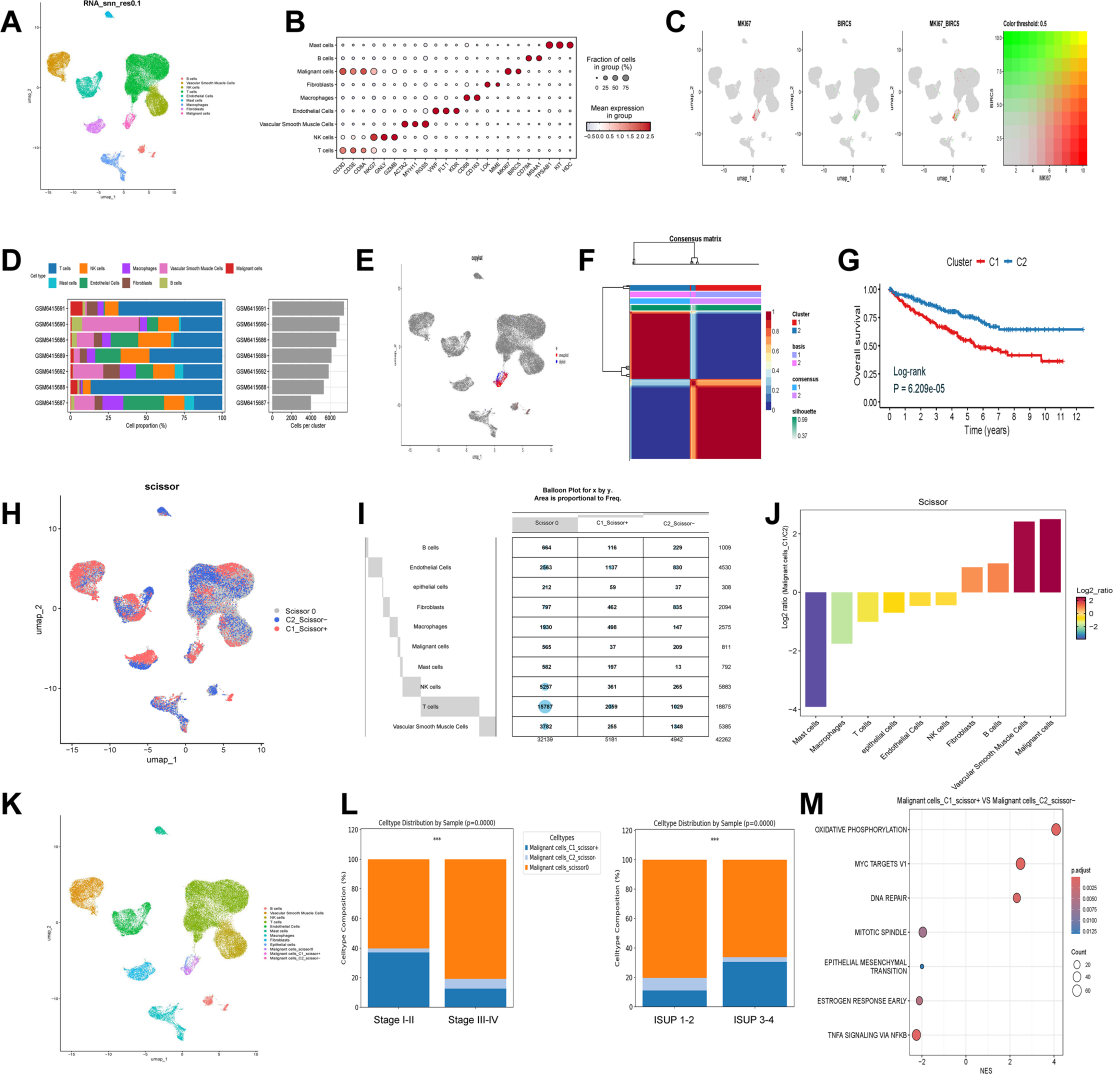
Single-cell annotation and identification of Ribosis-related malignant subtypes. **(A)** UMAP plot of nine clusters at resolution 0.1, corresponding to B cells, vascular smooth muscle cells, NK cells, T cells, endothelial cells, mast cells, macrophages, fibroblasts, and malignant cells. **(B)** Marker gene expression across the nine identified cell types. **(C)** Expression patterns of MKI67 and BIRC5 across single cells. **(D)** Proportions of different cell types across seven ccRCC samples. **(E)** Identification of diploid (non-malignant epithelial) and aneuploid (malignant) cells among malignant clusters. **(F)** NMF-based clustering of Ribosis-related gene expression profiles in the TCGA-ccRCC cohort. **(G)** Kaplan–Meier curves for patients with two Ribosis-related subtypes in TCGA-ccRCC. **(H)** Scissor-based mapping of bulk-defined Ribosis-related phenotypes (C1 and C2) to single cells. **(I)** Distribution of Ribosis phenotypes across cell clusters. **(J)** Ratios of C1_Scissor^+^ and C2_Scissor^-^ malignant cells. **(K)** UMAP showing 3 malignant, 1 epithelial, and 12 additional cell clusters. **(L)** Distribution of the three malignant subtypes across clinical parameters. **(M)** GSEA of hallmark pathways enriched in C1_Scissor^+^ versus C2_Scissor^-^ malignant cells. UMAP, uniform manifold approximation and projection; Log-rank, log-rank test; p.adjust, adjusted P value; ISUP, international society of urological pathology; MYC, myelocytomatosis; DNA, deoxyribonucleic acid; TNFA, tumor necrosis factor alpha; NFKB, nuclear factor kappa-B; ***p < 0.001.

The overall workflow of this study is outlined in **Figure 1**. Following quality control of the scRNA-seq data, a total of 42,262 cells were included in the analysis (**Supplementary Figure S1A, B**). Dimensionality reduction was first performed using PCA on the top 2,000 highly variable genes, and Harmony integration was applied to correct batch effects across multiple samples (**Supplementary Figure S1C, D**). Subsequently, unsupervised clustering of all cells was performed using UMAP, and clustering results at various resolutions were compared (**Supplementary Figure S1E**). A resolution of 0.1 was ultimately selected for downstream analyses (**Figure 2A**). Notably, across all tested resolutions, the spatial distribution of the malignant cell cluster remained highly stable, highlighting the distinctiveness of this population.

All cells were classified into nine distinct clusters, and cell types were manually annotated based on canonical markers. Marker gene expression across these clusters is shown in **Figure 2B**. MKI67 and BIRC5 were predominantly expressed in malignant cells and exhibited partial co-expression (**Figure 2C**). **Figure 2D** illustrates the cellular composition across seven ccRCC samples, showing that malignant cells represented only a minority of the total cell population.

To further distinguish malignant from non-malignant epithelial cells, we isolated the malignant cell cluster and applied the “CopyKAT” algorithm to infer chromosomal CNV. Aneuploid cells were annotated as malignant, whereas diploid cells were classified as epithelial cells (**Figure 2E**), resulting in the identification of 308 benign epithelial cells and 811 malignant cells. Using Ribosis-related genes, we performed NMF algorithm to identify transcriptional subtypes in the TCGA-ccRCC cohort. Clustering was optimal at k = 2 (**Supplementary Figure S1F**, **Figure 2F**), and Kaplan–Meier analysis revealed that patients in Cluster 1 (C1) exhibited significantly worse OS compared to those in Cluster 2 (C2) (**Figure 2G**).

To map these transcriptional phenotypes onto the scRNA-seq data, we applied the “Scissor” algorithm to deconvolve the bulk RNA-seq-defined C1 and C2 signatures onto single cells. Cells resembling the C1 phenotype were designated as C1_Scissor+, indicating potentially poorer prognostic features, whereas those associated with C2 were termed C2_Scissor–. Cells with no significant correlation were labeled Scissor0 (**Figure 2H**). Focusing on malignant cells, we identified 209 C1_Scissor+ and 37 C2_Scissor– cells—the highest proportions among all annotated clusters (**Figures 2I, J**). A comprehensive UMAP representation of these subtypes is shown in **Figure 2K**.

Importantly, we observed that the proportion of C1_Scissor+ malignant cells increased with tumor aggressiveness (tumor characteristics summarized in **Supplementary Table S5**), suggesting that Ribosis-based molecular subtypes may play a crucial role in tumor progression (**Figure 2L**). GSEA revealed significant enrichment of “OXIDATIVE PHOSPHORYLATION” (OXPHOS) and “MYC TARGETS V1” pathways in C1_Scissor+ malignant cells (**Figure 2M**).

### 3.2 Pseudotime analysis of malignant cell progression

**Figure 3:**
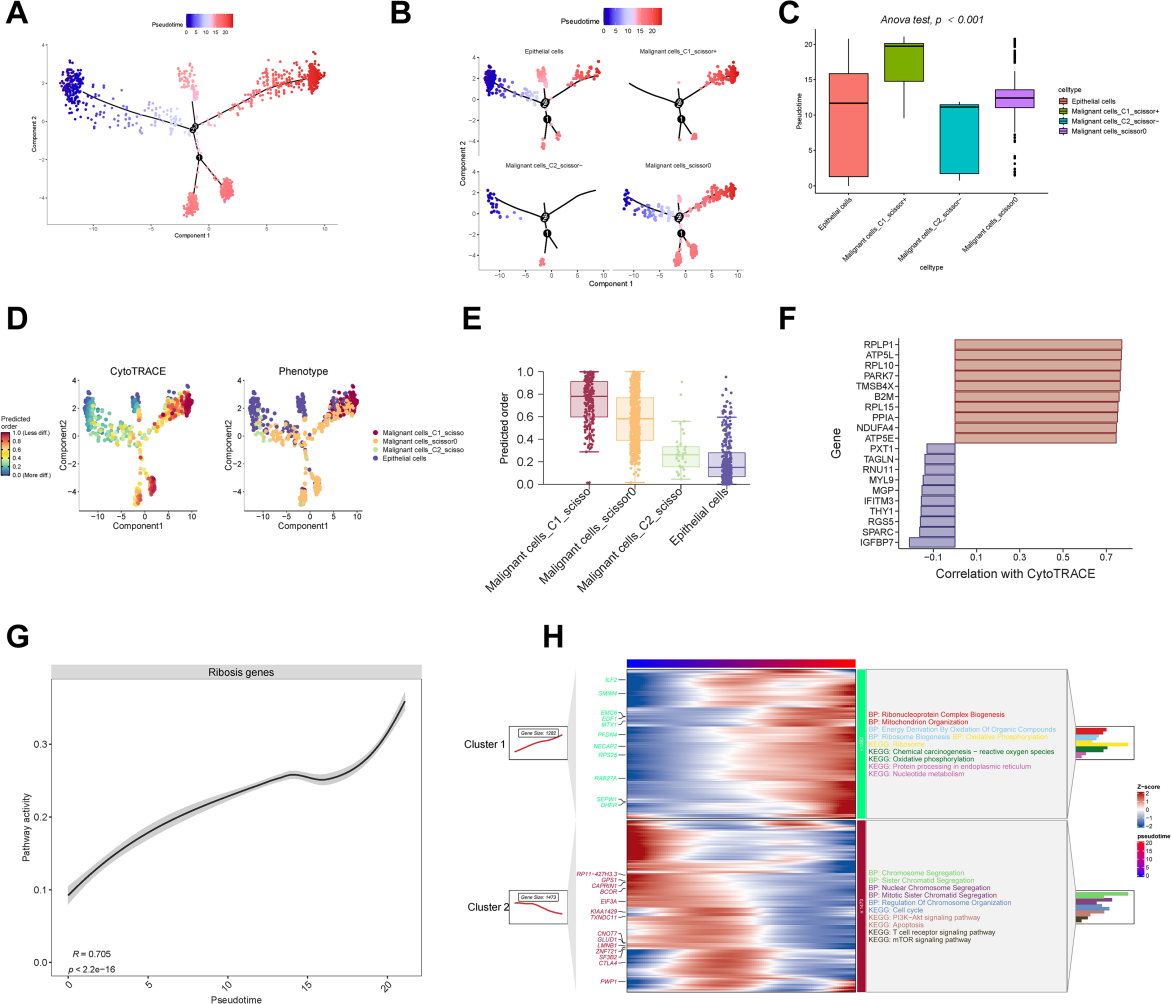
Pseudotime analysis and stemness evaluation. **(A–C)** Monocle trajectory and pseudotime inference of epithelial and malignant cells. **(D, E)** CytoTRACE-based quantification of cellular stemness. **(F)** Correlation between stemness scores and representative Ribosis genes. **(G)** Positive correlation between Ribosis activity and pseudotime progression. **(H)** ClusterGVis identifies early- and late-stage malignant cells based on pseudotime, with GO and KEGG enrichment of their associated pathways. ANOVA, analysis of variance; BP, biological process; KEGG, Kyoto Encyclopedia of Genes and Genomes; PI3K, phosphoinositide 3-kinase; mTOR, mammalian target of rapamycin.

To elucidate the evolutionary trajectory of malignant cells, we performed pseudotime analysis using the “Monocle” algorithm. Epithelial cells were specified as the origin of the trajectory (**Figure 3A**), based on their potential to undergo genetic alterations, aberrant activation, or epithelial-to-mesenchymal transition—biological events closely linked to malignant transformation (40, 41).

As shown in **Figures 3B, C**, C1_Scissor+ malignant cells predominantly occupied the terminal stages of the trajectory, indicating a late pseudotemporal state. In contrast, C2_Scissor– malignant cells were enriched at earlier stages. These pseudotime differences were statistically significant across subtypes. We further validated developmental status using “CytoTRACE” algorithm (**Figure 3D**), which confirmed that C1_Scissor+ malignant cells exhibited the highest stemness, while C2_Scissor– malignant cells showed the lowest (**Figure 3E**). Cancer cells possess strong self-renewal and differentiation capacities, and those at more advanced stages of malignant progression typically exhibit enhanced stemness (42), consistent with our findings.

Cellular stemness was positively correlated with expression of Ribosis-related genes RPLP1, RPL10, and RPL15 (**Figure 3F**), and overall Ribosis activity also increased significantly along pseudotime (**Figure 3G**), suggesting a key role for Ribosis in malignant progression. “ClusterGVis” algorithm-based trajectory clustering revealed two dominant branches (**Figure 3H**): Cluster 1 cells, enriched at the pseudotime endpoint, were associated with Ribosis and OXPHOS pathways, while Cluster 2 cells, enriched at early stages, were associated with cell cycle-related processes.

### 3.3 Metabolic profiling of malignant cells at the single-cell level

**Figure 4:**
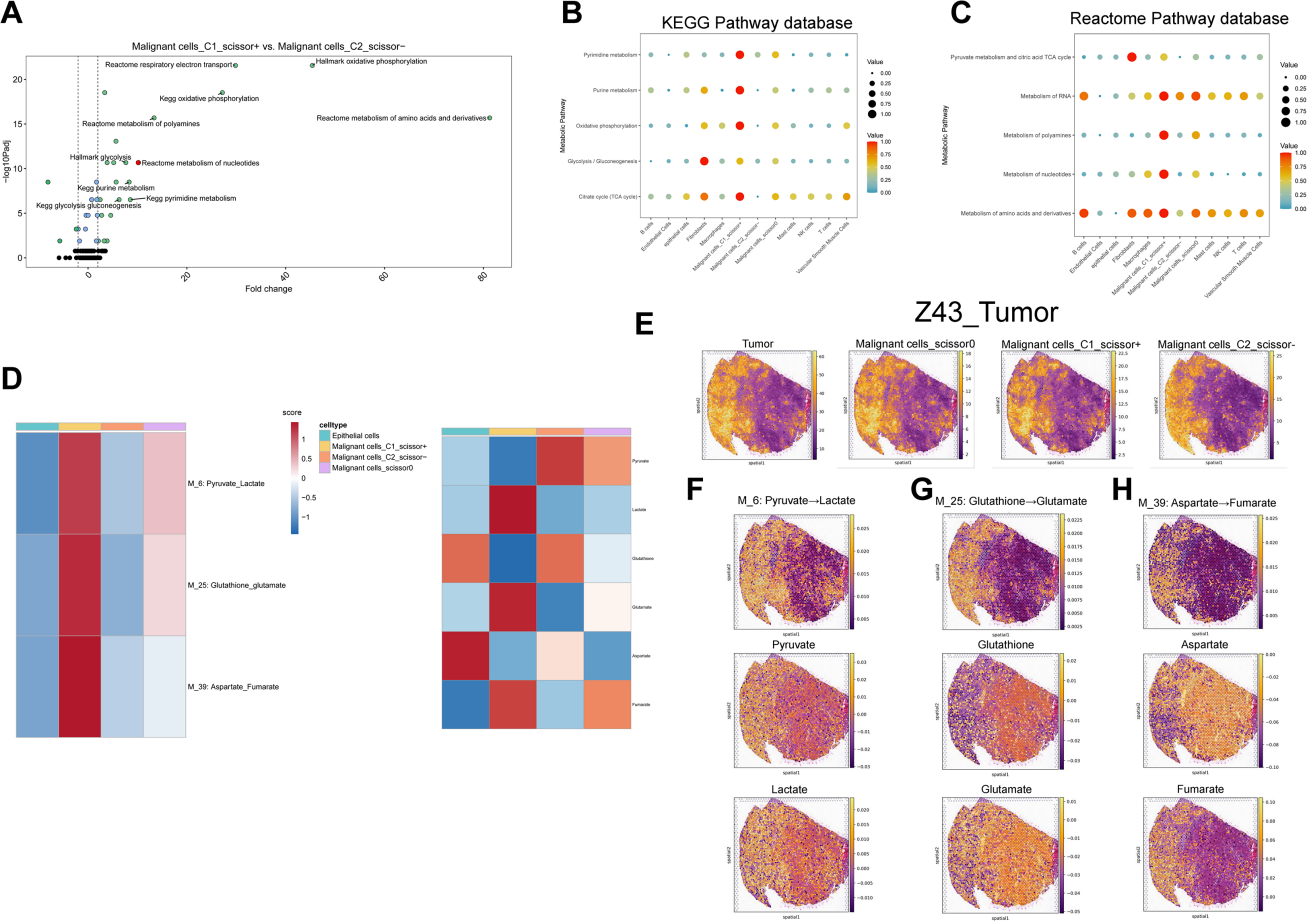
Single-cell metabolic pathway and metabolite analysis. **(A)** Top 10 differentially enriched metabolic pathways between C1_Scissor^+^ and C2_Scissor^-^ malignant cells (SPCA). **(B, C)** Enrichment of five core metabolic pathways across cell types using KEGG and Reactome databases. **(D)** scFEA-based flux and abundance estimates for intermediate modules and metabolites across epithelial and malignant subtypes. **(E)** Spatial localization of tumor core regions and three malignant subtypes in samples “Z43”. **(F-H)** Spatial flux and abundance patterns for three metabolic modules and their corresponding metabolites. Padj, adjusted P value; TCA, tricarboxylic acid; M_6, metabolic module 6; Pyruvate_Lactate, pyruvate-to-lactate conversion pathway.

Aberrant metabolism contributes to cancer initiation, progression, metastasis, therapeutic resistance, and maintenance of cancer stemness via multiple mechanisms. A deeper understanding of these metabolic alterations may reveal fundamental aspects of tumor biology and inform the development of novel therapeutic strategies (43). Using curated KEGG and Reactome pathway datasets, we applied “SPCA” algorithm to analyze metabolic differences between malignant cell subtypes. The analysis revealed striking differences between C1_Scissor+ and C2_Scissor– malignant cells, with the top 10 most divergent pathways primarily related to energy, carbohydrate, nucleotide, and protein metabolism (**Figure 4A**). We next quantified the activity of these pathways using the “scMetabolism” algorithm (**Figures 4B, C**). Among all malignant subtypes, C1_Scissor+ malignant cells exhibited the highest overall metabolic activity, followed by Scissor0 malignant cells, while C2_Scissor– malignant cells displayed the weakest activity.

To explore metabolic flux and metabolite abundance, we further applied the “scFEA” algorithm across epithelial cells and the three malignant subtypes. In C1_Scissor+ malignant cells, intermediate pathways such as glycolysis–tricarboxylic acid cycle, serine metabolism, and aspartate metabolism were significantly upregulated, contrasting sharply with the patterns observed in C2_Scissor– malignant cells (**Supplementary Figures S2A, B**). We highlighted three of the most distinctive metabolic pathways in C1_Scissor+ malignant cells and visualized their associated metabolite abundances (**Figure 4D**), where scores above zero indicated metabolite accumulation and scores below zero indicated depletion.

Interestingly, C1_Scissor+ and C2_Scissor– malignant cells exhibited almost opposite patterns in metabolite abundance. The most notable difference was observed in the “M_6: Pyruvate_Lactate” axis, where pyruvate was markedly depleted, and lactate accumulated in C1_Scissor+ malignant cells—a pattern reversed in the other malignant subtypes (**Figure 4D**). This phenomenon reflects the well-established Warburg effect, in which cancer cells preferentially convert pyruvate to lactate rather than fueling mitochondrial OXPHOS. Lactate accumulation acidifies the tumor microenvironment (TME), promoting tumor invasion and metastasis while impairing immune function and facilitating immune evasion (44). These results suggest that the pyruvate–lactate axis may be a key contributor to the poor prognosis of C1_Scissor+ malignant cells and highlight Ribosis-associated metabolic reprogramming—particularly the pyruvate–lactate pathway—as a potential therapeutic target for combined metabolic and immunologic intervention.

### 3.4 Spatial metabolic landscape of malignant cells

To investigate the spatial distribution of malignant subtypes and their metabolic activity, we employed the “cell2location” algorithm to project scRNA-seq-defined cell states onto stRNA-seq data. This analysis was performed on two tumor specimens, “R114T” and “Z43T,” and revealed spatial distributions of the three malignant cell subtypes that closely mirrored tumor regions. Notably, C1_Scissor+ malignant cells occupied a slightly broader spatial area compared to C2_Scissor– malignant cells (**Figure 4E**, **Supplementary Figure S3A**). Subtype proportions were highly similar between the two samples (**Supplementary Figure S4A**), suggesting minimal inter-sample heterogeneity and justifying integrated metabolic analysis across both datasets.

We next applied the “scFEA” algorithm to evaluate metabolic flux and metabolite abundance within the spatial context. We focused on the same three intermediate pathways and six metabolites highlighted in **Figures 4D** (**Figures 4F-H**, **Supplementary Figures S3B–D**). Apart from the “M_6: Pyruvate_Lactate” pathway, spatial features of the other two pathways and associated metabolites remained consistent with those observed in single-cell data, suggesting that C1_Scissor+ malignant cells are the dominant contributors to these metabolic activities.

**Figure 5:**
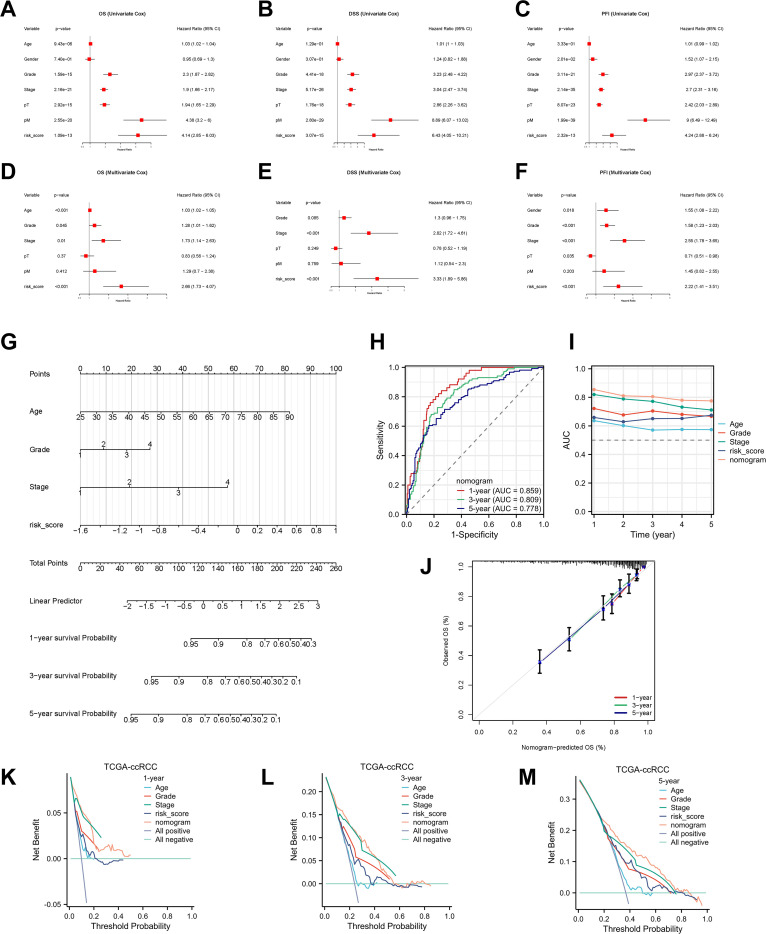
Development and validation of the nomogram. **(A–F)** Univariable and multivariable Cox analyses identifying independent prognostic factors for OS, DSS, and PFI. **(G)** Nomogram integrating RBRS, age, grade, and stage. **(H, I)** ROC and C-index comparisons of nomogram versus clinical features. **(J)** Calibration curves. **(K–M)** DCA for 1-, 3-, and 5-year OS. 95% CI, 95% confidence interval; OS, overall survival; DSS, disease-specific survival; PFI, progression-free interval; pT, pathological tumor stage; pM, pathological metastasis stage; AUC, area under the curve; TCGA, The Cancer Genome Atlas; ccRCC, clear cell renal cell carcinoma.

For the “M_6: Pyruvate_Lactate” pathway, pyruvate abundance was generally positive across tumor regions in the spatial transcriptomic data, indicating metabolite accumulation. When interpreted alongside **Figure 5E**, this suggests that the combined accumulation of pyruvate by C2_Scissor– and Scissor0 malignant cells may exceed the consumption by C1_Scissor+ malignant cells, given their overlapping spatial distributions—ultimately resulting in pyruvate accumulation in spatial data.

### 3.5 Construction of a Ribosis-relatedsignature using machine learning

**Figure 6:**
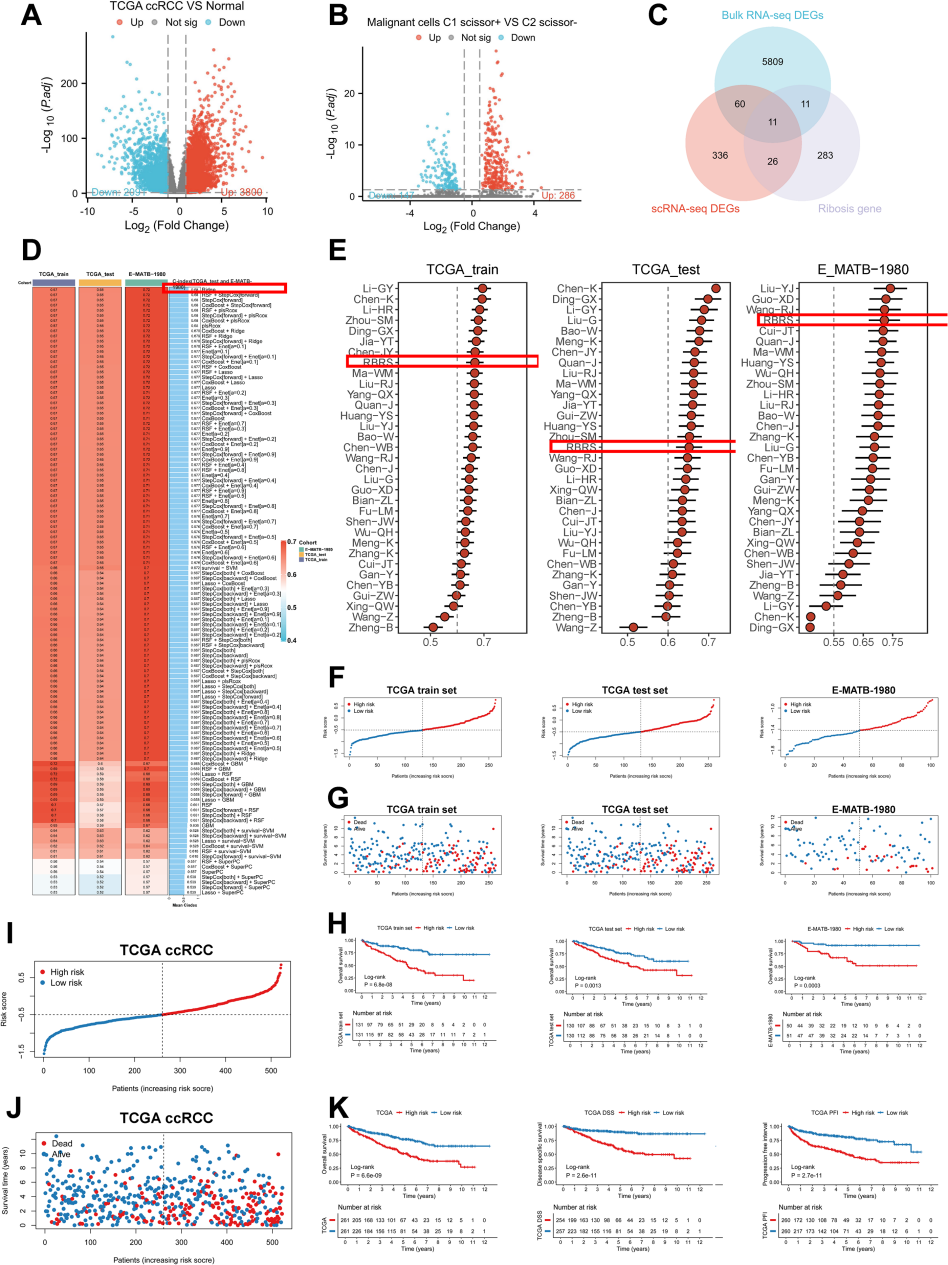
Construction of the RBRS prognostic model. **(A)** Volcano plot of DEGs between tumor and normal samples in TCGA-ccRCC. **(B)** Volcano plot of DEGs between C1_Scissor^+^ and C2_Scissor^-^ malignant cells. **(C)** Intersection of DEGs from bulk RNA-seq, scRNA-seq, and Ribosis-related genes. **(D)** Performance of 118 machine learning models in TCGA training, test, and E-MATB-1980 cohorts (C-index). **(E)** Comparison of RBRS with 32 published ccRCC prognostic signatures. **(F–H)** RBRS score, survival status, and Kaplan–Meier curves in three cohorts. **(I, J)** Combined TCGA cohorts. **(K)** Kaplan–Meier analysis of OS, DSS, and PFI based on RBRS scores. TCGA, The Cancer Genome Atlas; ccRCC, clear cell renal cell carcinoma; DEGs, differentially expressed genes; RNA-seq, RNA sequencing; RBRS, ribosome biogenesis-related signature; DSS, disease-specific survival; PFI, progression-free interval.

To develop a Ribosis-related prognostic signature, we first performed differential gene expression analysis between tumor and normal tissues in the TCGA-ccRCC cohort using the “DESeq2” algorithm. A total of 3,800 upregulated and 2,091 downregulated genes were identified (**Figure 6A**, **Supplementary Table S6**). In parallel, differential expression between the C1_Scissor+ and C2_Scissor– malignant cells were assessed using the “FindMarkers” function, yielding 286 upregulated and 147 downregulated genes (**Figure 6B**, **Supplementary Table S7**). By intersecting these DEGs with Ribosis-related genes, 11 candidates were preliminarily identified (**Figure 6C**). We next conducted univariate Cox regression analysis to evaluate the prognostic relevance of these 11 genes. Given the small number of candidates, a relaxed p-value threshold of 0.1 was used. Multivariate Cox regression further refined the selection, ultimately identifying five prognostically significant genes (**Supplementary Table S8**). To enhance clinical applicability, we developed a prognostic model based on these five genes using 118 combinations of 10 machine learning algorithms. The TCGA-ccRCC cohort was randomly divided into training and test sets at a 1:1 ratio (**Supplementary Table S9**). The training set was used for feature selection and model construction, while the model was validated in both the test set and an external cohort (E-MATB-1980). C-index values were computed across all datasets (**Figure 6D**). Among all models, Ridge regression yielded the highest average C-index (0.68) across the validation cohorts and was thus selected to define the RBRS. The final RBRS risk score was computed using the following formula: Risk score = 0.1886866 × RPL38 + 0.3353690 × RPS2 – 0.4759008 × RPS14 + 0.3914829 × RPS19 – 0.5451182 × RPS28.

These five Ribosis genes exhibited spatial expression patterns highly concordant with tumor core regions in the “R114T” and “Z43T” spatial transcriptomic samples (**Supplementary Figures S4B, C**). Moreover, their expression levels were significantly positively correlated with pseudotime progression (**Supplementary Figure S4D**), consistent with previous findings that aberrant Ribosis activity is associated with late-stage tumors (45).

To benchmark the RBRS model, we compared its prognostic performance with 32 published ccRCC signatures retrieved from PubMed (**Supplementary Table S10**). Notably, RBRS displayed relatively stable and robust performance across all datasets (**Figure 6E**). Using the RBRS formula, individual risk scores were calculated for each patient across datasets. Patients were stratified into high- and low-risk groups based on the median score, revealing a progressive increase in mortality with rising risk score (**Figures 6F, G**). Kaplan–Meier survival analysis confirmed that patients in the high-risk group exhibited significantly poorer prognosis (P < 0.05) (**Figure 6H**). Similar trends were observed in the merged TCGA training and test cohorts (**FigureS 5I, J**). Furthermore, high RBRS scores were consistently associated with inferior outcomes across OS, disease-specific survival (DSS), and progression-free interval (PFI) (P < 0.001) (**Figure 6K**). The area under the curve (AUC) values demonstrated stable performance across datasets: in the TCGA training set, AUC at 1, 3, and 5 years were 0.679, 0.661, and 0.712, respectively; in the test set, 0.673, 0.655, and 0.645; in the merged cohort, 0.671, 0.657, and 0.678; and in the external E-MATB-1980 cohort, 0.704, 0.720, and 0.705 (**Supplementary Figure S5A**).

We further investigated the relationship between RBRS and clinical characteristics in the TCGA-ccRCC cohort (**Supplementary Table S11**). Significant differences were observed between high- and low-risk groups in terms of OS, survival status, histological grade, clinical stage, pT, pN, and pM classifications (**Supplementary Figures S5B, C**). Due to the high proportion of missing pN data and the small number of patients with lymph node metastases, pN stage was excluded from further subgroup analyses. Patients with pT3–4, M0, Stage III–IV, and Grade III–IV exhibited significantly higher RBRS scores than those with pT1–2, M1, Stage I–II, and Grade I–II, respectively (**Supplementary Figures S5D–G**). Except in the Grade I–II subgroup, high RBRS scores consistently correlated with worse OS across all clinical subgroups (**Supplementary Figures S5H–M**). Collectively, these findings indicate that RBRS is closely associated with poor prognosis in ccRCC.

### 3.6 Development and validation of an integrated prognostic model for ccRCC

To assess the independent prognostic value of RBRS, both univariate and multivariate Cox regression analyses were performed. In univariate analysis, RBRS was significantly associated with OS (hazard ratio [HR] = 4.14; 95% confidence interval [CI] = 2.85–6.03), DSS (HR = 6.43; 95% CI = 4.05–10.21), and PFI (HR = 4.24; 95% CI = 2.88–6.24) (**Figures 5A–C**). In multivariate analysis, RBRS remained an independent prognostic factor for OS (HR = 2.66; 95% CI = 1.03–4.07), DSS (HR = 3.33; 95% CI = 1.89–5.86), and PFI (HR = 2.22; 95% CI = 1.41–3.51) (**Figures 5D–F**).

To further extend the clinical utility of RBRS, we constructed an integrated prognostic nomogram incorporating RBRS along with age, tumor grade, and clinical stage (**Figure 5G**). The nomogram demonstrated robust predictive performance for 1-, 3-, and 5-year OS with AUC of 0.859, 0.809, and 0.778, respectively (**Figures 5H, I**). Calibration curves revealed excellent concordance between predicted and observed survival probabilities (**Figure 5J**), while DCA confirmed that the nomogram provided superior net clinical benefit compared to individual prognostic factors at all time points (**Figures 5K–M**).

To facilitate broad clinical application, we deployed the nomogram as a web-based tool using the Shiny platform (https://drxie2018510136.shinyapps.io/shinyapp/), enabling users to input clinical features and RBRS scores to obtain dynamic, individualized survival predictions for ccRCC patients.

### 3.7 Genomic mutation landscape

**Figure 7:**
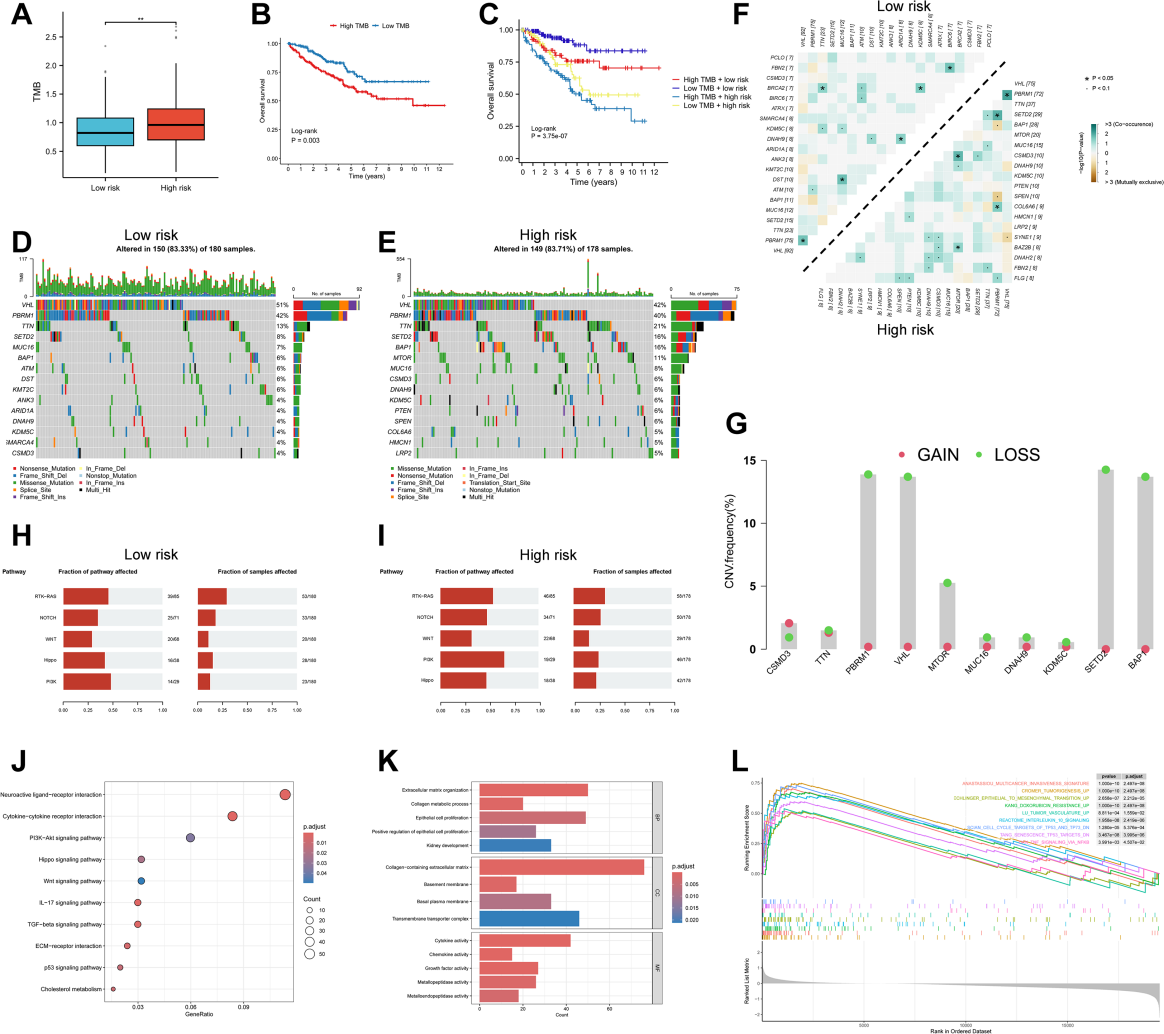
Genomic landscape and pathway enrichment in high- vs low-risk RBRS groups. **(A)** TMB distribution by risk. **(B, C)** Survival curves by TMB and combined TMB/RBRS status. **(D, E)** Mutation waterfall plots. **(F)** Co-occurrence and mutual exclusivity of top mutated genes. **(G)** CNV frequency of top 10 mutated genes. **(H, I)** Oncogenic pathway enrichment. **(J)** KEGG; **(K)** GO; **(L)** GSEA. TMB, tumor mutation burden; Log-rank, log-rank test; CNV, copy number variation; RTK, receptor tyrosine kinase; RAS, rat Sarcoma; PI3K, phosphoinositide 3-kinase; IL-17, interleukin-17; TGF, transforming growth factor; ECM, extracellular matrix; p53, protein 53; BP, biological process; MF, molecular function; CC, cellular component; p.adjust, adjusted P value; TP53, tumor protein 53; TP73, tumor protein 73; DN; down; TNF, tumor necrosis factor; NFKB, nuclear factor kappa-B; **p < 0.01 .

Intratumoral heterogeneity, a hallmark of cancer, is primarily driven by the accumulation of somatic mutations and plays a central role in tumor progression, therapeutic resistance, and disease relapse (46). Using the “maftools” algorithm, we evaluated TMB across ccRCC patients and found that high-risk individuals exhibited significantly elevated TMB scores compared to the low-risk group (P < 0.01), along with worse survival outcomes (P = 0.003) (**Figures 7A, B**). Stratified survival analysis integrating both TMB and RBRS risk scores revealed that patients with the “High TMB + high risk” combination had the poorest prognosis (P = 3.75e-07) (**Figure 7C**).

We next examined the mutational profiles across risk groups. Canonical mutations in ccRCC—such as VHL, SETD2, PBRM1, and BAP1—were recurrently observed (**Figures 7D, E**), and have been previously described as trunk mutations contributing to genomic instability and impaired DNA repair (47). Among the top six most frequently mutated genes in the high-risk group, TTN, SETD2, BAP1, and MTOR showed significantly increased mutation frequencies (P < 0.05), while VHL and PBRM1 did not differ significantly between groups (P > 0.05) (**Supplementary Figure S6A**).

Additionally, we analyzed patterns of co-occurrence and mutual exclusivity among the top 20 mutated genes across risk groups. The high-risk group demonstrated a greater degree of co-occurring mutations (**Figure 7F**). CNV, another driver of tumorigenesis, can alter oncogene or tumor suppressor gene expression (48). CNV analysis of the top 10 frequently mutated genes revealed that PBRM1, VHL, SETD2, and BAP1 had the highest rates of copy number loss, while copy number gains were less prominent (**Figure 7G**). These four genes are well-established tumor suppressors in ccRCC (47), and loss of one or both alleles often leads to complete functional inactivation. For instance, deletion of the VHL gene due to CNV loss is a frequent event in ccRCC (49).

Expression and survival analyses using bulk RNA-seq data revealed that these four tumor suppressor genes were significantly downregulated in tumor tissues compared to normal controls (**Supplementary Figures S6B–E**). Moreover, lower expression of SETD2, PBRM1, and BAP1 was associated with worse prognosis, while VHL expression showed no significant survival impact (**Supplementary Figure S6F–I**). These results suggest that CNV loss may alter tumor suppressor gene expression and influence patient outcomes.

Pathway enrichment of mutated genes revealed associations with several oncogenic pathways (**Figures 7H, I**). NOTCH and PI3K signaling pathways were enriched among high-risk patients (P < 0.05), whereas other pathways showed no significant group differences (P > 0.05) (**Supplementary Figure S6J**). Prior studies have shown that targeting the Notch pathway, both *in vitro* and *in vivo*, can suppress ccRCC growth (50). Similarly, PI3K signaling is a major oncogenic driver and therapeutic target in ccRCC, strongly implicated in disease progression (51). These findings suggest that the prognostic divergence between RBRS-defined subgroups may be closely linked to distinct genomic mutation landscapes.

### 3.8 Pathway enrichment analysis across risk subgroups

To explore the molecular mechanisms underlying differential gene expression between RBRS-defined risk subgroups, we performed enrichment analysis using the “clusterProfiler” algorithm. KEGG pathways were enriched for cancer-related processes, immune regulation, inflammation, metabolism, and TME remodeling (**Figure 7J**). ccRCC is characterized by complex interactions and dysregulation across multiple signaling pathways, including PI3K–Akt, Hippo, Wnt, TGF-β, and p53, which are known to drive cancer progression (52).

Extracellular matrix remodeling is a hallmark of tumor growth and metastasis. Extracellular matrix components and their receptors not only facilitate tumor dissemination but also serve as a mechanical barrier that hinders therapeutic penetration (53). Cholesterol metabolic reprogramming is a distinct feature of ccRCC. Studies have shown that ccRCC cells heavily rely on exogenous cholesterol uptake rather than endogenous biosynthesis. Targeting SCARB1, a key cholesterol transporter, can induce cell cycle arrest, apoptosis, and PI3K/AKT pathway inhibition in ccRCC (54).

GO analysis revealed key biological processes including renal epithelial growth, tumor development, extracellular matrix (ECM) remodeling, and cytokine signaling (**Figure 7K**). GSEA further showed that high-risk patients were enriched for gene signatures involved in tumor invasion and initiation, angiogenesis, inflammation, chemoresistance, and p53 pathway suppression (**Figure 7L**). Given the well-documented roles of these pathways in cancer biology, detailed discussion is omitted here. Collectively, these findings reinforce the connection between RBRS and oncogenic biological and metabolic processes in ccRCC.

### 3.9 Immune microenvironment and immune checkpoint expression across risk subgroups

**Figure 8:**
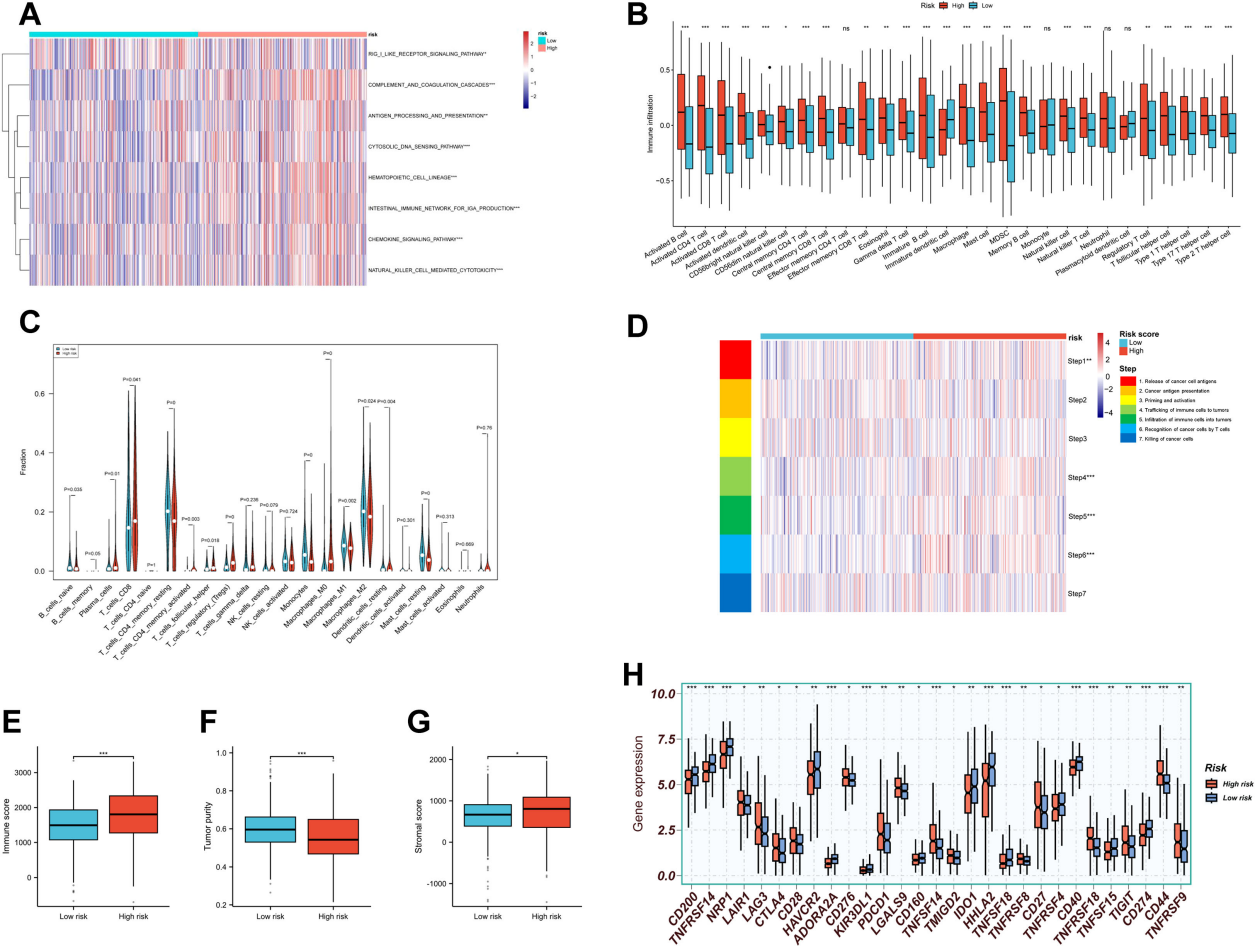
Tumor immune microenvironment landscape in RBRS subgroups. **(A)** Heatmap of immune pathway activity. **(B, C)** Infiltration of immune cells quantified via ssGSEA and CIBERSORT. **(D–F)** Immune score, tumor purity, and stromal score comparisons. **(G)** Anti-cancer immunity cycle activity. **(H)** Immune checkpoint expression. RIG-I, retinoic acid-inducible gene I; DNA, deoxyribonucleic acid; IGA, immunoglobulin A; MDSC, myeloid-derived suppressor cell; ns, not significant; *p < 0.05; **p < 0.01; ***p < 0.001.

The immunosuppressive nature of the TME is a major barrier to effective immunotherapy. Understanding how to reshape the immune microenvironment is crucial for overcoming resistance and identifying new therapeutic targets (55). We used the “GSVA” algorithm to evaluate 15 immune-related pathways. Except for the RIG-I-like receptor pathway, high-risk patients exhibited stronger activation across pathways related to innate immunity, adaptive immune response, immune cell development, and inflammatory cytotoxic signaling (**Figure 8A**).

Using “ssGSEA” algorithm, we assessed the correlation between immune cell infiltration and risk score. High-risk patients displayed increased infiltration of adaptive immune cells such as activated CD4^+^ and CD8^+^ T cells (**Figure 8B**). However, suppressive immune cells—including myeloid-derived suppressor cells (MDSCs) and regulatory T cells (Tregs)—were also more abundant in this group. Similar trends were observed using CIBERSORT analysis: CD8^+^ T cells and memory-activated CD4^+^ T cells coexisted with Tregs in the high-risk group (**Figure 8C**). These findings suggest that high-risk tumors may exist in a state of “co-activation and co-suppression” within the immune microenvironment.

Given the complexity of the TME, immune status cannot be evaluated solely by immune cell abundance (56). We therefore applied TIP analysis to examine seven steps of the cancer–immunity cycle (**Figure 8D**). The high-risk group showed enhanced activity in antigen release (Step 1) and immune cell infiltration (Steps 4–6), but no corresponding increase in final tumor cell killing (Step 7), suggesting ineffective immune clearance.

We also used the “ESTIMATE” algorithm to compute immune scores, tumor purity, and stromal scores across subgroups (**Figures 8E-G**). High-risk patients exhibited higher immune scores, indicating a more active immune environment, but had lower tumor purity and higher stromal scores, implying greater infiltration of non-malignant components such as fibroblasts, mesenchymal stem cells, and ECM. These stromal elements cooperatively contribute to tumor progression, metastasis, and resistance (57), highlighting the need for combined immunotherapy and stroma-targeting strategies.

Finally, expression of immune checkpoint molecules—including PDCD1, CTLA4, TIGIT, and LAG3—was significantly elevated in the high-risk group (**Figure 8H**), indicating potential targets for immune-based combination therapies.

### 3.10 Drug sensitivity prediction and validation of RBRS-related genes

**Figure 9:**
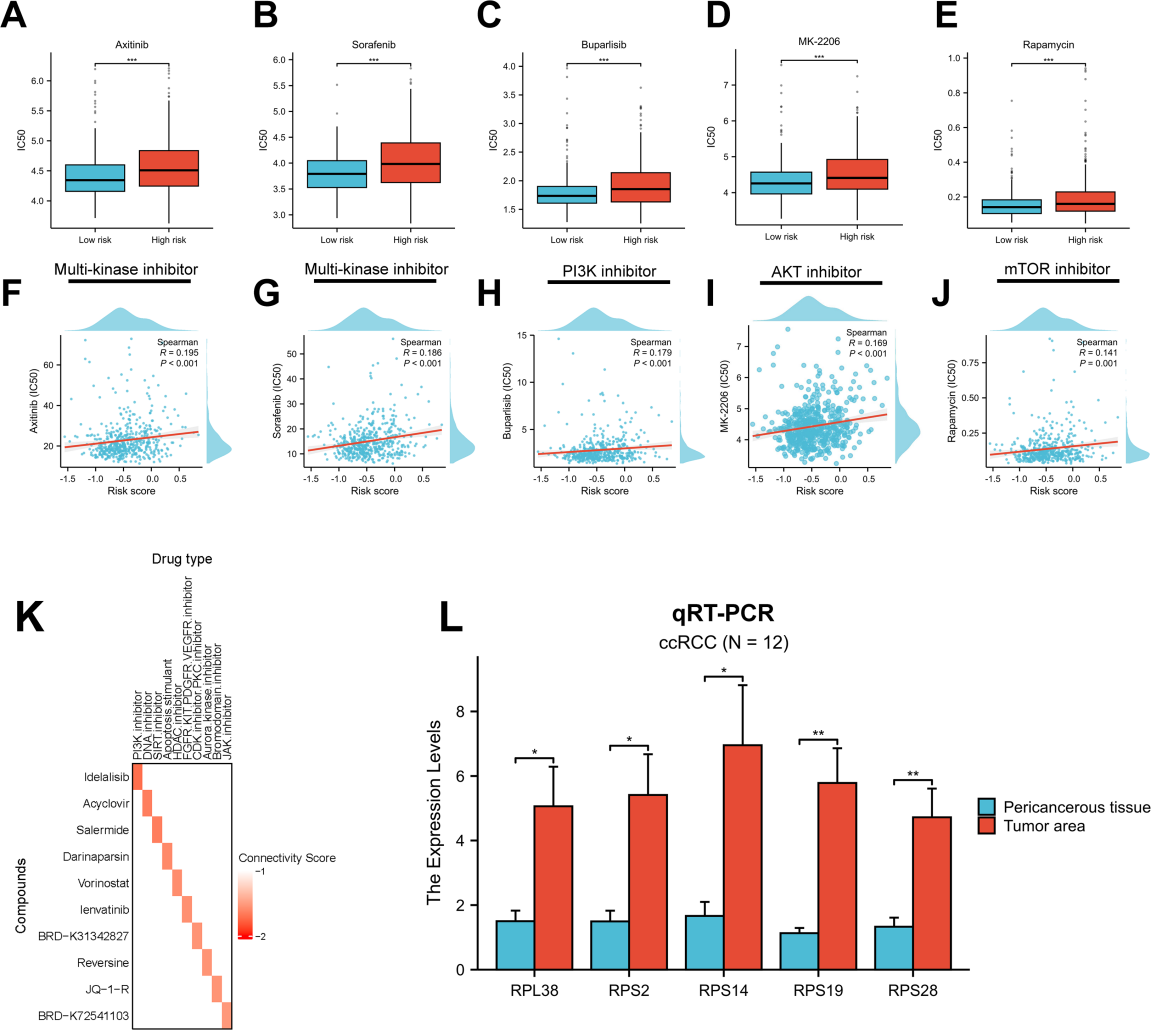
Drug sensitivity and gene expression validation. **(A–E)** Sensitivity to five targeted therapies by RBRS subgroup. **(F–J)** Correlation between RBRS score and drug IC50. **(K)** Top 10 candidate drugs predicted by cMap. **(L)** qRT-PCR validation of five RBRS genes in paired tumor and adjacent tissues from ccRCC patients. IC50, half-maximal inhibitory concentration; PI3K, phosphoinositide 3-kinase; mTOR, mammalian target of rapamycin; DNA, deoxyribonucleic acid; SIRT, sirtuin; HDAC, histone deacetylase; FGFR, fibroblast growth factor receptor; KIT, tyrosine kinase; PDGFR, platelet-derived growth factor receptor; VEGFR, vascular endothelial growth factor receptor; CDK, cyclin-dependent kinase; PKC, protein kinase C; JAK, Janus kinase; ccRCC, clear cell renal cell carcinoma; qRT-PCR, quantitative real-time PCR; *p < 0.05; **p < 0.01; ***p < 0.001.

Therapeutic resistance remains a major challenge in cancer treatment, largely due to the dynamic and heterogeneous nature of the TME (58). To evaluate drug response across RBRS subgroups, we used the “oncoPredict” algorithm. All significantly different compounds are listed in **Supplementary Figure S6K**. In the context of advanced RCC, first-line therapies often include multi-kinase inhibitors (MKIs) and mammalian target of rapamycin (mTOR) inhibitors (4). Our analysis revealed that high-risk patients exhibited greater resistance to MKIs and PI3K/AKT/mTOR pathway inhibitors (**Figures 9A–E**), with drug IC50 values positively correlated with RBRS scores (**Figures 9F–J**).

To identify candidate compounds with potential to reverse the high-risk gene expression profile, we submitted the top 150 upregulated and 150 downregulated genes from each risk group to the cMap database (**Supplementary Table S12**). Ten compounds with the lowest connectivity scores and defined mechanisms of action were selected (**Figure 9K**). These agents may inhibit ccRCC progression and offer therapeutic opportunities for targeting malignant transformation.

Lastly, we validated the expression of the five RBRS genes (RPL38, RPS2, RPS14, RPS19, and RPS28) using qRT-PCR on tumor and adjacent normal samples from 12 ccRCC patients at Meizhou Hospital. All five genes were significantly upregulated in tumor tissues (**Figure 9L**), consistent with TCGA-ccRCC transcriptomic data (**Supplementary Table S6**), supporting their potential functional relevance in ccRCC development and progression.

## 4 Discussion

### 4.1 Biological context and novelty

ccRCC, the most prevalent subtype of renal cancer, remains clinically challenging due to its high heterogeneity and therapeutic resistance (2, 4). Dysregulated Ribosis has recently emerged as a central oncogenic mechanism in multiple malignancies, driving tumorigenesis via onco-ribosome-mediated metabolic reprogramming and selective translation of oncogenic transcripts (7–9). However, its molecular characteristics and clinical significance in ccRCC have not been systematically investigated.

In this study, we integrated multi-omics data—including bulk RNA-seq, scRNA-seq, and stRNA-seq—to comprehensively elucidate the associations between Ribosis and malignant evolution, metabolic rewiring, and immune microenvironment remodeling in ccRCC. Moreover, we developed a RBRS prognostic model using advanced machine learning strategies. RBRS demonstrated robust predictive performance and translational potential, offering new mechanistic insights and a precision medicine framework for ccRCC.

### 4.2 Ribosis and malignant progression

Aberrant Ribosis is not merely a passive consequence of tumorigenesis, but a key driver of cancer progression (59). In the TCGA-ccRCC cohort, patients with high Ribosis-related gene expression (Cluster 1) had significantly worse OS, suggesting its role in malignant progression and its potential as a biomarker for patient stratification. At the single-cell level, Ribosis activity was markedly elevated in C1_Scissor+ malignant cells, which were enriched at the terminal end of the pseudotime trajectory and exhibited enhanced stemness features.

We observed a positive correlation between cellular stemness and expression of several Ribosis-related genes (e.g., RPLP1, RPL10, RPL15), as well as a progressive increase in Ribosis activity along pseudotime. These findings suggest that Ribosis may promote ccRCC progression by sustaining stem-like states and facilitating malignant evolution—consistent with the aggressive features of advanced-stage tumors, which exhibit enhanced stemness and hyperactive Ribosis (42, 45).

In terminal-stage malignant cells, we also noted a concurrent upregulation of OXPHOS. GSEA revealed significant enrichment of OXPHOS and MYC target pathways in C1_Scissor+ relative to C2_Scissor– malignant cells, suggesting a possible synergistic role in driving tumor progression. MYC is a well-known upstream regulator of Ribosis that enhances ribosomal DNA transcription and ribosomal protein production, thereby promoting cancer cell growth (12). OXPHOS, on the other hand, supplies energy for Ribosis and is also implicated in T cell dysfunction and immune resistance under hypoxic conditions in ccRCC (60, 61). This metabolic-oncogenic interplay may help explain the persistence of aggressive phenotypes and resistance to therapy in metabolically reprogrammed tumors (62). By contrast, early-stage malignant cells were enriched for cell division-related pathways, consistent with the hyperproliferative phenotype of early tumor evolution driven by clonal competition (63).

### 4.3 Ribosis and metabolic reprogramming

Cancer cells undergo metabolic reprogramming to meet the high biosynthetic, energetic, and immune-evasive demands of tumor growth (43). As the central engine of protein synthesis, Ribosis directly contributes to remodeling metabolic networks to support sustained tumor proliferation and invasion (7). In our study, C1_Scissor+ malignant cells with high Ribosis activity exhibited elevated expression across key metabolic pathways, including glycolysis, amino acid, and nucleotide metabolism. These cells also showed pronounced pyruvate depletion and lactate accumulation, hallmarks of the Warburg effect. Lactate acidification of the TME can impair T cell function, thereby promoting immune escape and metastasis (44).

Ribosis requires abundant biosynthetic precursors and energy, necessitating reprogrammed metabolism. Cancer cells often enhance aerobic glycolysis to generate adenosine triphosphate and intermediates to support macromolecule synthesis (44). Notably, ribosomal stress can itself feedback into metabolism, promoting secondary mutations and adaptation (64). The enrichment of multiple metabolic pathways in C1_Scissor+ malignant cells reflect this adaptation to a high-biosynthesis state.

Metabolic products like lactate not only reflect reprogramming but also shape immune microenvironments. Importantly, Ribosis and metabolism are bidirectionally regulated: nutrient-sensing and growth factor pathways (e.g., PI3K–AKT–mTOR) activate rRNA synthesis when metabolite levels are sufficient, whereas tumor suppressors like p53 inhibit Ribosis under energy stress or ribosomal damage (59). Thus, malignant cells often exist in a “high-metabolism, high-synthesis” state, wherein metabolic rewiring both enables and reinforces Ribosis and tumor aggressiveness.

### 4.4 Ribosis and immune microenvironment remodeling

The ccRCC immune microenvironment is highly complex, posing significant challenges to therapeutic efficacy (65). Our results indicate that high RBRS risk scores are associated not with immune exclusion, but with a state of “coexistent immune activation and suppression.” While high-risk tumors exhibited increased infiltration of effector CD4^+^ and CD8^+^ T cells, they also harbored elevated levels of immunosuppressive cells such as Tregs and MDSCs. This composition suggests the presence of antitumor immunity that is simultaneously dampened by suppressive elements, leading to T cell exhaustion and immune evasion (65, 66). Immune evasion in ccRCC is primarily driven by T cell exhaustion (67), and may also involve tumor-mediated recruitment and alteration of immune cells to establish an immunosuppressive microenvironment, a common mechanism observed in many cancers (68). Tregs are known to be major inhibitors of effective immune responses in ccRCC, suppressing CD8^+^ T cell activation via cytokines such as transforming growth factor-β and interleukin 10, and depleting Interleukin 2 to block clonal expansion (69, 70). MDSCs further aggravate immune suppression by producing reactive oxygen species, arginase, and promoting M2-like macrophage polarization (71). TIP analysis revealed that while high-risk tumors are active in antigen release and T cell recruitment (Steps 1 and 4–6), they are impaired in tumor-killing capacity (Step 7). This “active initiation but exhausted termination” pattern highlights the dominance of immunosuppressive signaling in ccRCC TME (67). Furthermore, ECM rigidity and the recruitment of tumor-associated macrophages contribute to constructing an immune-suppressive niche, further hindering therapeutic response (62, 72). These structural and cellular features underscore the critical need to decode Ribosis-associated immune remodeling.

Given the immunogenic yet evasive nature of ccRCC, immune checkpoint inhibitors have gained traction (73). Expression of checkpoint molecules—PDCD1 (PD-1), CTLA-4, TIGIT, and LAG3—was significantly elevated in the high-risk group. PD-1 inhibitors (e.g., nivolumab, pembrolizumab) and CTLA-4 inhibitors (e.g., ipilimumab) are already approved for advanced ccRCC (4). TIGIT suppresses antitumor immunity by expanding Tregs and promoting M2 macrophages (74). A phase II trial of the TIGIT inhibitor (tiragolumab) in advanced RCC is underway (NCT05805501). LAG3, linked to T cell exhaustion via binding to MHC-II or FGL1, is another promising target (75). A phase II trial showed that nivolumab plus LAG3 blockade (relatlimab) achieved comparable efficacy to nivolumab plus ipilimumab with improved safety in advanced RCC (76), and two additional trials (NCT05148546 and NCT06708949) are ongoing. Notably, when evaluating new anticancer drug candidates, it is essential to carefully assess potential toxicity to ensure both safety and efficacy in clinical translation (5).

### 4.5 Translational potential of the RBRS model

In the era of precision medicine, robust biomarkers are critical for stratifying risk and guiding ccRCC treatment. The RBRS model, derived from Ribosis-related gene expression, demonstrated high prognostic accuracy (AUC of 0.859, 0.809, and 0.778 at 1, 3, and 5 years, respectively), underscoring its clinical potential. To enhance usability, we developed a dynamic online tool (https://drxie2018510136.shinyapps.io/shinyapp/) enabling individualized risk assessment to support clinical decision-making.

In addition to its prognostic value, the RBRS model holds promise as a clinical decision-support tool for guiding therapeutic strategies in ccRCC. To further translate this potential into practice, RBRS-based risk stratification could be integrated with established clinicopathological parameters and molecular markers to optimize individualized treatment selection. Specifically, patients with high RBRS scores—who tend to exhibit immunosuppressive tumor microenvironments—may benefit more from combination strategies involving immune checkpoint inhibitors and anti-angiogenic agents, such as pembrolizumab plus axitinib, rather than monotherapies. RBRS scoring could thus serve as a triage tool to prioritize patients for combination regimens early in their treatment course.

Moreover, incorporating RBRS into future clinical trial designs could refine patient selection criteria and improve response rate predictability for emerging therapeutics. Our cMap analysis further revealed several small-molecule compounds with potential anti-Ribosis activity. Although these candidates are still at the computational prediction stage, future efforts should include their functional validation in preclinical models to assess mechanisms of action, therapeutic efficacy, pharmacokinetics, and toxicity profiles. Particular attention should be paid to repurposed drugs with known safety margins, which may expedite translation into early-phase trials. Ultimately, integrating RBRS-based profiling with therapeutic response data could pave the way for a more tailored and responsive approach to managing ccRCC, advancing RBRS from a prognostic biomarker to a clinically actionable precision oncology tool.

### 4.6 Adjunctive therapeutic prospects beyond Ribosis

While the RBRS model provides a robust framework for risk stratification and therapeutic guidance, its integration with emerging adjunctive strategies may further enhance clinical benefit in ccRCC. The rapid development of novel physical therapies has opened new avenues for ccRCC treatment. Physical stimulation modalities—including ionizing radiation, phototherapy, electricity, magnetic fields, and ultrasound—have been reported to modulate the TME by remodeling vasculature, altering ECM composition, and activating immune responses, thereby enhancing tumor antigen exposure and immune cell infiltration. These effects have shown superior efficacy compared to immune monotherapies (77). In particular, low-frequency magnetic fields disrupt actin polymerization dynamics, impairing cytoskeletal integrity and selectively inhibiting tumor cell migration, while concurrently promoting local immune infiltration (78).

In the field of nanomedicine, surface-engineered and hybrid nanoparticles are enabling deeper tumor penetration and more precise delivery of therapeutic payloads. These features may indirectly support the future delivery of anti-Ribosis compounds while reducing systemic toxicity (79, 80). Additionally, systemic interventions such as regular physical activity have been shown to reprogram tumor-associated metabolic and immune networks (81), potentially counteracting the immunosuppressive and hypoxic niches characteristic of high-RBRS tumors.

Although direct evidence in ccRCC remains limited, the TME-modulating properties of these strategies may complement Ribosis-targeted metabolic and immunologic interventions. Their conceptual compatibility with the RBRS framework warrants further mechanistic investigation and broader integration into combination regimens or supportive care paradigms.

### 4.7 Limitations

Despite the comprehensive insights into Ribosis and the development of a robust prognostic model, several limitations must be acknowledged. First, our analyses were based on publicly available datasets (e.g., TCGA, GEO), and validation in large, real-world multicenter cohorts is still lacking. The number of scRNA-seq and stRNA-seq samples was also limited, potentially underrepresenting ccRCC heterogeneity. Second, further optimization of RBRS detection methods is needed for application to routine pathology specimens. Third, the mechanistic links between Ribosis and tumor progression were only preliminarily validated via bioinformatics and qRT-PCR. Due to experimental and technical constraints, this study lacks systematic *in vitro* and *in vivo* functional validation. Future investigations should incorporate mechanistic studies to further elucidate the causal role of Ribosis in ccRCC progression.

## 5 Conclusions

This study systematically characterized the expression and functional significance of Ribosis in ccRCC and developed a high-performance RBRS prognostic model. The RBRS offers a novel tool for risk stratification and precision therapy in ccRCC and lays a foundation for future investigations into Ribosis-driven mechanisms and targeted interventions.

## Data Availability

Publicly available datasets were analyzed in this study. This data can be found here: The datasets analyzed in this study are publicly available from the following sources: bulk RNA-seq data for the TCGA-ccRCC cohort were obtained from the TCGA portal (https://portal.gdc.cancer.gov/); bulk RNA-seq data for the E-MTAB-1980 cohort were retrieved from the ArrayExpress database (https://www.ebi.ac.uk/arrayexpress/); single-cell RNA-seq data from 7 ccRCC samples were acquired from the GEO database (https://www.ncbi.nlm.nih.gov/geo/); and spatial transcriptomics data from 2 ccRCC samples were downloaded from the ZENODO repository (https://zenodo.org/records/8063124).

## Supplementary material

Supplementary Figure 1 Single-cell QC and NMF clustering. **(A, B)** Pre- and post-QC for seven single-cell samples. **(C, D)** Before and after PCA batch correction. **(E)** UMAPs at various resolutions. **(F)** Selection of optimal NMF clusters. mt, mitochondrial genes; ribo, ribosomal genes; hb, hemoglobin genes; PCA, principal component analysis; RNA, ribonucleic acid; snn, shared nearest neighbor; res, resolution; NMF, negative matrix factorization

Supplementary Figure 2 Panoramic overview of single-cell metabolic pathways and metabolite profiles. **(A, B)** Flux and abundance estimates for intermediate modules and metabolites across epithelial and malignant subtypes.

Supplementary Figure 3 Spatial distribution of metabolic modules and metabolites. **(A)** Spatial localization of tumor core regions and three malignant subtypes in samples “R114”. **(B–D)** Spatial flux and abundance patterns for three metabolic modules and their corresponding metabolites. M_6, metabolic module 6; Pyruvate_Lactate, pyruvate-to-lactate conversion pathway

Supplementary Figure 4 Spatial and pseudotime expression of RBRS genes. **(A)** Distribution of three malignant subtypes in spatial transcriptomics. **(B, C)** Spatial localization of five RBRS genes. **(D)** Correlation of gene expression with pseudotime. ns, not significant; R114_T, R114 tumor; Z43_T, Z43 tumor

Supplementary Figure 5 Evaluation of the RBRS model. **(A)** ROC curves for predicting 1-, 3-, and 5-year OS across datasets. **(B, C)** Clinical feature distribution between RBRS risk groups. **(D–G)** Risk scores across clinical subgroups (pT, pM, stage, grade). **(H–M)** Kaplan–Meier curves of OS stratified by RBRS and clinical subgroups. TCGA, The Cancer Genome Atlas; ccRCC, clear cell renal cell carcinoma; AUC, area under the curve; RS, risk score; OS, overall survival; pT, pathological tumor stage; pN, pathological node stage; pM, pathological metastasis stage; Log-rank, log-rank test; *, p < 0.05; **, p < 0.01; ***, p < 0.001

Supplementary Figure 6 Mutation differences and drug responses in RBRS subgroups. **(A)** Differential mutation profiles. **(B–I)** Expression and survival associations for four high-frequency CNV genes. **(K)** Drug sensitivity across RBRS subgroups. **(L)** Enrichment of oncogenic pathways in mutated genes from high- and low-risk patient groups. OR, odds ratio; 95% CI, 95% confidence interval; RTK, receptor tyrosine kinase; RAS, rat Sarcoma; PI3K, phosphoinositide 3-kinase; TPM, transcripts per million; Log-rank, log-rank test; ***, p < 0.001
