# Supplementary figures and images for: Multi-omics analysis reveals the role of ribosome biogenesis in malignant clear cell renal cell carcinoma and the development of a machine learning-based prognostic model
Source: Front Immunol. 2025 Jun 26;16:1602898. doi: 10.3389/fimmu.2025.1602898 (PMC12240986; doi:10.3389/fimmu.2025.1602898)

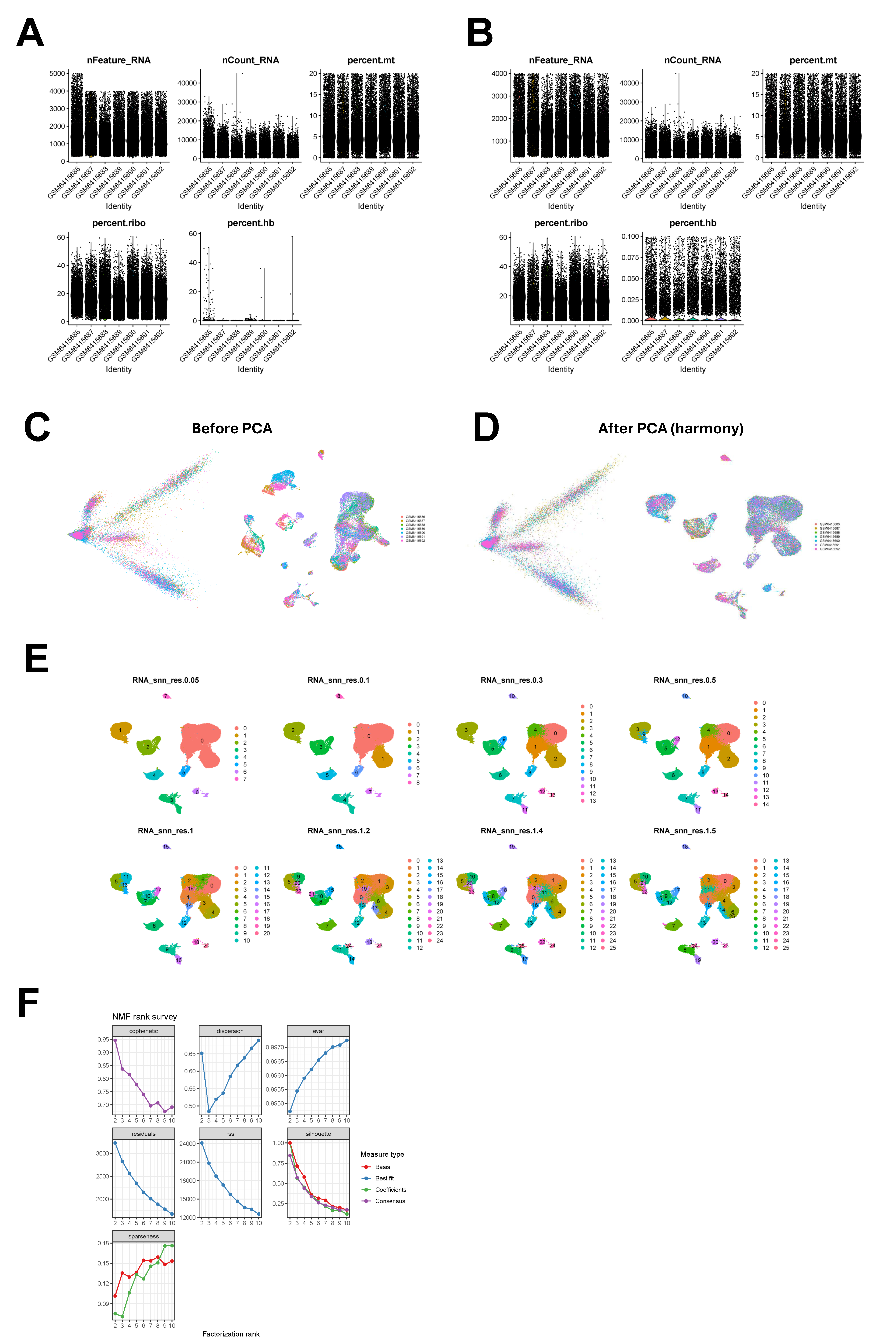

Supplement: Supplementary Figure 1 — Single-cell QC and NMF clustering. (A, B) Pre- and post-QC for seven single-cell samples. (C, D) Before and after PCA batch correction. (E) UMAPs at various resolutions. (F) Selection of optimal NMF clusters. mt, mitochondrial genes; ribo, ribosomal genes; hb, hemoglobin genes; PCA, principal component analysis; RNA, ribonucleic acid; snn, shared nearest neighbor; res, resolution; NMF, negative matrix factorization [file Image1.tif]

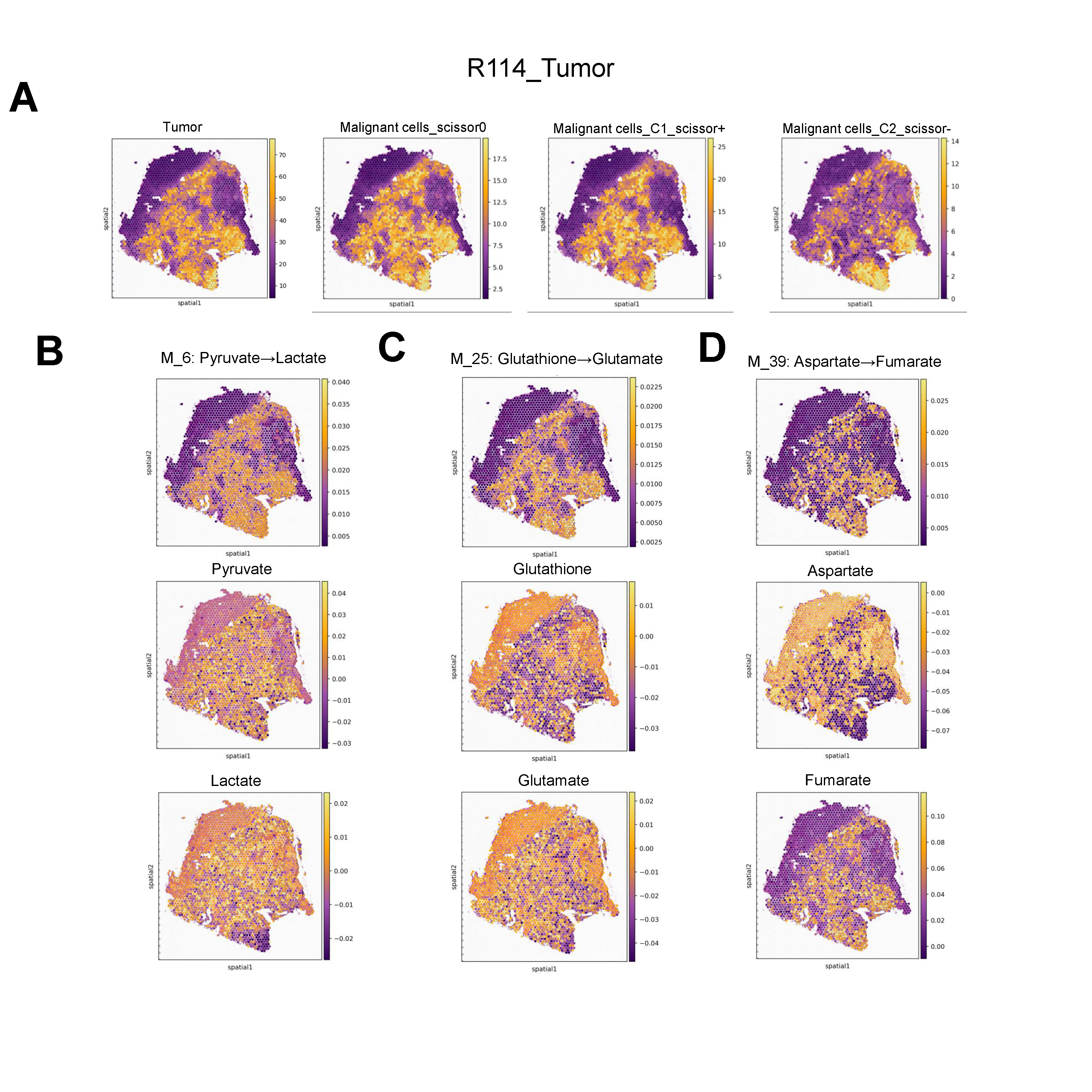

Supplement: Supplementary Figure 3 — Spatial distribution of metabolic modules and metabolites. (A) Spatial localization of tumor core regions and three malignant subtypes in samples “R114”. (B–D) Spatial flux and abundance patterns for three metabolic modules and their corresponding metabolites. M_6, metabolic module 6; Pyruvate_Lactate, pyruvate-to-lactate conversion pathway [file Image3.tif]

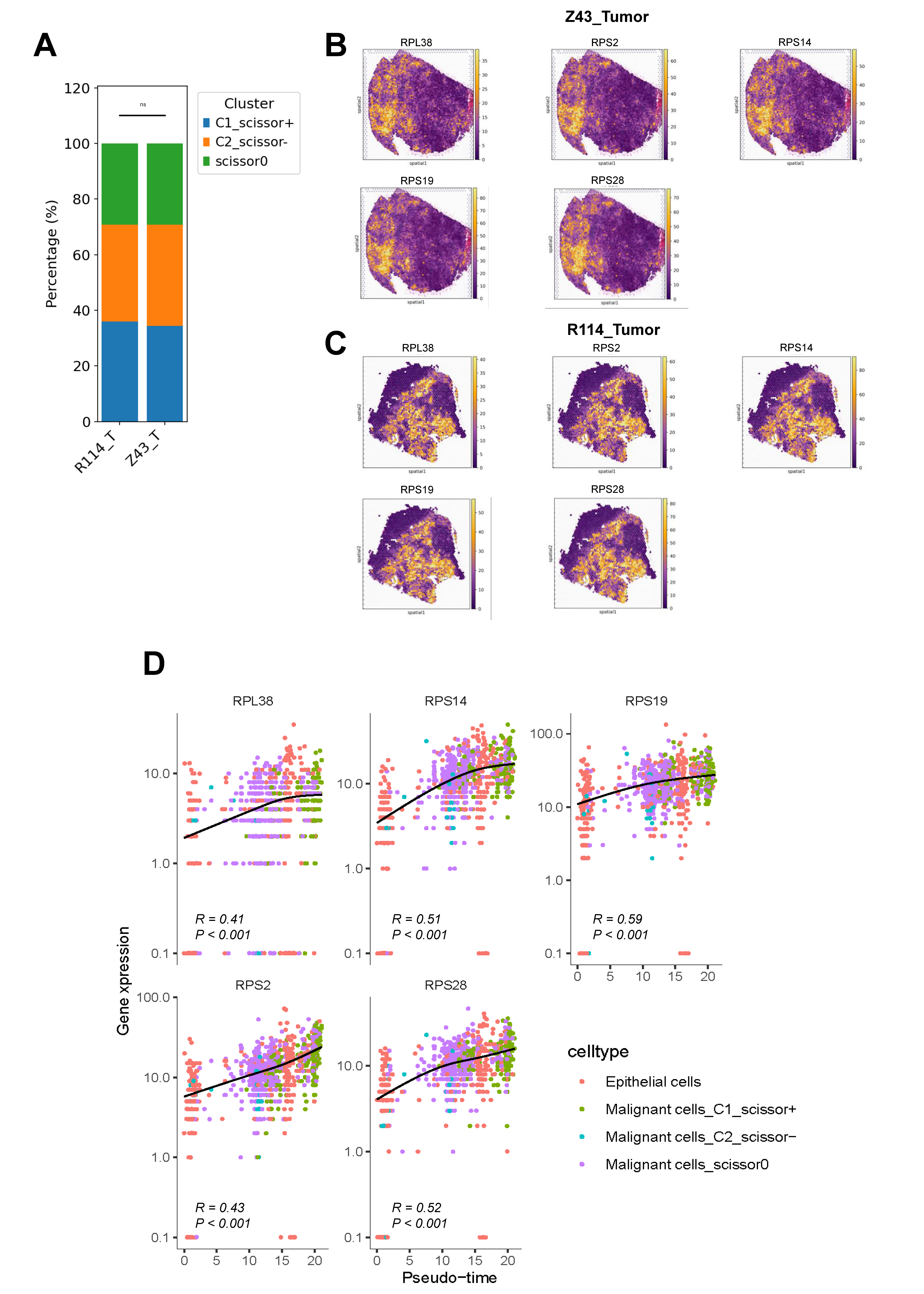

Supplement: Supplementary Figure 4 — Spatial and pseudotime expression of RBRS genes. (A) Distribution of three malignant subtypes in spatial transcriptomics. (B, C) Spatial localization of five RBRS genes. (D) Correlation of gene expression with pseudotime. ns, not significant; R114_T, R114 tumor; Z43_T, Z43 tumor [file Image4.tif]
